# School bullying results in poor psychological conditions: evidence from a survey of 95,545 subjects

**DOI:** 10.3389/fpsyg.2024.1279872

**Published:** 2024-01-24

**Authors:** Na Zhao, Shenglong Yang, Qiangjian Zhang, Jian Wang, Wei Xie, Youguo Tan, Tao Zhou

**Affiliations:** ^1^Key Laboratory in Software Engineering of Yunnan Province, Yunnan University, Kunming, China; ^2^Big Data Research Center, University of Electronic Science and Technology of China, Chengdu, China; ^3^Faculty of Information Engineering and Automation, Kunming University of Science and Technology, Kunming, China; ^4^Chengdu Happy Xiaoqing Intelligent Technology Co. LTD, Chengdu, China; ^5^Department of Psychological Counseling, Zigong Fifth People's Hospital, Zigong, China; ^6^Computational Education Lab, SeekingTao Tech. Inc., Chengdu, China

**Keywords:** school bullying, mental health, adolescents, logistic regression analysis, less developed city

## Abstract

To investigate whether bullying and psychological conditions are correlated, this study analyzed a survey of primary and secondary school students from Zigong City, Sichuan Province. A total of 95,545 students completed a personal information questionnaire, the Multidimensional Peer-Victimization Scale (MPVS), and eight other scales pertaining to various psychological problems. The data showed that 68,315 (71.5%) participants experienced school bullying at varying degrees, indicating the prevalence of bullying among adolescents. The chi-square tests revealed a strong correlation between school bullying and psychological conditions. This correlation was further explored through multivariate logistic regression, showing that students who experienced mild bullying had a 3.10 times higher probability of emotional and behavioral problems, 4.06 times higher probability of experiencing prodromal symptoms of mental illness, 4.72 times higher probability of anxiety, 3.28 times higher probability of developing post-traumatic stress disorder (PTSD), 4.07 times higher probability of poor sleep quality, 3.13 times higher probability of internet addiction, 2.18 times higher probability of poor mental health, and 3.64 times higher probability of depression than students who did not experience bullying. The corresponding probabilities for students who experienced severe bullying were 11.35, 17.35, 18.52, 12.59, 11.67, 12.03, 4.64, and 5.34 times higher, respectively. In conclusion, school bullying and psychological conditions are significantly correlated among primary and secondary school students, and the more severe the bullying, the higher the probability to suffer from psychological problems.

## 1 Introduction

The teenage years are a critical period for physical growth and development, mental maturity, personality formation, and the attainment of scientific and cultural knowledge. Psychological problems, such as depression, can lead to several negative outcomes in teenagers, including poor academic performance, alcohol abuse, and suicide (Xu et al., 2022). The reasons for poor psychological conditions are complex, involving personal, family, and school factors. Schools, as large gathering places for primary and secondary school students, allow students to frequently interact with each other. These interactions, including school bullying, can significantly impact students' psychological wellbeing (Hamel et al., 2021).

The increasing number of cases of school bullying in the twenty-first century has drawn widespread social attention to related studies (Olweus, 2013). Early studies showed that psychological conditions are strongly related to school bullying, which is defined as continued attacks by teachers or students on certain students within the school campuses and surrounding areas (Hymel and Swearer, 2015). Traditional bullying can be divided into four categories: physical victimization, verbal victimization, social manipulation, and attacks on property (Mynard and Joseph, 2000). School bullying is prevalent among teenagers globally: the proportion of teenagers affected by school bullying across different countries varies between 4.8 and 45.2% (Craig et al., 2009).

Researchers have conducted in-depth studies on school bullying. For example, Cosma et al. (2020) compared the cross-national trends of school bullying to examine the differences between cyberbullying and traditional bullying. Chudal et al. (2021) investigated the prevalence of bullying and its various types among 21,688 13–15-year-old adolescents in developing countries. Pengpid and Peltzer (2019) studied the relationship between school bullying and psychological problems among adolescents in Southeast Asian countries. Pörhölä et al. (2020) surveyed 8,497 students from several countries to examine the differences between various types of school bullying. Existing research has further explored the impact of school bullying on psychological problems. Chen and Elklit (2018) analyzed 4,051 bullied adolescents, revealing the relationship between school bullying and post-traumatic stress disorder (PTSD). Meanwhile, Zhou et al. (2015) systematically studied the correlation between school bullying and poor sleep quality. Some researchers have proposed various theories to explain the observed correlation between school bullying and poor psychological conditions. Estévez et al. (2009) claimed that students who are bullied in school may experience long-term negative behaviors from their peers, which will cause them to face greater psychological stress and be more likely to experience social isolation, and this social isolation will increase their risk of developing psychological symptoms like anxiety, depression, and so on. Arslan et al. (2021) pointed out that school bullying may damage the self-esteem and self-confidence of victimized students, and continued exposure to negative behaviors from peers may cause them to have a negative view of themselves, thereby affecting their mental health. Nazir and Nesheen (2015) showed that school bullying will cause academic problems of both perpetrators and victims, and then academic problems and psychological problems will interact with each other, forming a vicious circle. However, the existing body of literature on the relationship between school bullying and psychological problems has shortcomings, including small sample sizes, lack of attention paid to adolescents in developing countries, lack of comparative studies on various types of bullying, and lack of analysis on multiple dimensions of psychological problems.

The present study aimed to fill this gap in the literature by surveying a large sample of students from developing countries. Furthermore, to offer detailed findings, this study sought to cover the four types of school bullying and distinguish between different degrees and different types of school bullying.

## 2 Methods

### 2.1 Research procedure

The research procedure can be roughly divided into four steps: (i) Implementing Questionnaire Surveys, (ii) Collecting and Preprocessing Data, (iii) Analyzing Data, and (iv) Drawing Conclusion. More detailed description is as follows.

#### 2.1.1 Implementing questionnaire surveys

We have collaborated with the government of Zigong city to implement the extensive questionnaire surveys for 158 schools. For each school, we have trained a few teachers to distribute and retrieve surveys, as well as to guide and supervise students filling in the surveys. Those surveys include one basic information questionnaire, one validated scale for school bullying, and eight validated scales for psychological health status.

#### 2.1.2 Collecting and preprocessing data

As all analyzed questions are choice questions (the true and false questions can be considered as binary choice questions), we have scanned the survey and automatically obtain the digital records by using a standard optical character recognition (OCR) technique. The manual cross-check show a 100% accuracy since such OCR task is very easy and standard. We have cleaned the data before further analysis by removing surveys with missing values and outliers.

#### 2.1.3 Analyzing data

We applied the group analysis method to intuitively see the differences in psychological health status of students in different groups (e.g., being bullied versus not being bullied), and then we performed the chi-square test to measure the statistical significance of the correlation between school bullying and psychological health status.

#### 2.1.4 Drawing conclusions

Based on the results from the data, we draw conclusions about the relationship between school bullying and psychological problems, and discuss the interpretation of statistical significance and the practical meanings of the conclusions.

### 2.2 Participants

This study involved a questionnaire survey of 95,545 primary and secondary school students. The sample included 27,128 (29%) primary school students from 71 primary schools, 43,124 (45%) junior high school students from 73 junior high schools, and 25,203 (26%) senior high school students from 14 senior high schools in Zigong City, Sichuan Province, China. The participants were aged 6–22 years (average age: 13.47 years), with primary school students mainly 6–12 years, junior high school students mainly 13–15 years, and senior high school students mainly 16–18 years.

### 2.3 Ethical statement

All participants were informed of the purpose of the study, and participation was voluntary. Each participant and her/his parents signed written consent forms. To protect the participants' privacy, the survey data were anonymized.

### 2.4 Questionnaire survey data

#### 2.4.1 Basic information

Using the Basic Information Survey Questionnaire, the study collected data on participants' age, school, living environment, family situation, and family environment, to analyze the reasons for their psychological problems from personal and family perspectives.

#### 2.4.2 School bullying

The Multidimensional Peer-Victimization Scale (MPVS; Mynard and Joseph, 2000) was used to investigate the prevalence of school bullying, including physical and verbal victimization, property damage, and insults from peers during the participants' growth process. The types of victimization included hitting, insulting, teasing, slandering, exclusion, spreading rumors, and so on. The participants were required to truthfully fill out the MPVS to reflect their experiences of school bullying.

#### 2.4.3 Psychological problems

We assessed participants' overall psychological well-being by measuring their psychological health status across eight dimensions. These dimensions corresponded to validated scales, including the Strengths and Difficulties Questionnaire—Short Version (SDQ–S; Goodman et al., 2000) for emotional and behavioral problems, the Prodromal Questionnaire—16 Items (PQ–16; Miller et al., 1999) for psychotic risk, the Generalized Anxiety Disorder 7-Item Scale (GAD-7; Tennant et al., 2007) for anxiety, the Children's Revised Impact of Event Scale-13 Items (CRIES-13; Perrin et al., 2005) for stress response, the Pittsburgh Sleep Quality Index (PSQI; Buysse et al., 1989; Carpenter and Andrykowski, 1998) for sleep quality, the nine-item Internet Gaming Disorder Scale—Short Form (IGDS9-SF; Pontes and Griffiths, 2015; Monacis et al., 2016) for internet addiction, the Patient Health Questionnaire—9 item (PHQ-9; Kroenke et al., 2001) for depression, and the Warwick-Edinburgh Mental Well-Being Scale (WEMWBS; Tennant et al., 2007) for mental well-being. After participants completed the aforementioned scales, we evaluated participants' psychological conditions based on the scale scores.

#### 2.4.4 Questionnaire reliability

Cronbach's alpha is a well-known coefficient to measure the internal reliability of a scale (Tavakol and Dennick, 2011), based on the overall variance and the variance of each item. Specifically, Cronbach's alpha is calculated as


$$\alpha =\frac{k}{k-1}\left(1-\frac{\overset{k}{\underset{i=1}{\sum }}\sigma_{i}^{2}}{\sigma_{T}^{2}}\right)$$

where *k* is the number of questions in the scale, $\sigma_{i}^{2}$ is the variance of the ith question, and $\sigma_{T}^{2}$ is the variance of all results. To test the internal consistency of the questionnaire, we first calculated the Cronbach's alpha coefficient for each scale. For the MPVS scale, which is used to investigate the status of school bullying, α = 0.797. For the eight scales used to investigate the mental health status, the corresponding coefficients are SDQ-S (0.831), PQ-16 (0.763), GAD-7 (0.861), CRIES-13 (0.829), PSQI (0.857), IGDS9-SF (0.752), PHQ-9 (0.849), and WEMWBS (0.734). Every scale's alpha is higher than 0.7, showing an acceptable reliability.

We utilized the Harman's single-factor test (Fuller et al., 2016) operation to quantify the common method bias present in our data. Harman's single-factor test is a statistical method used to test common method bias. It tests whether there is a single factor that explains most of the variance in the data by performing factor analysis on the data. If such a single factor exists, then it may be caused by common method bias. We first use the principal component analysis (PCA) by treating each question in the questionnaire as a factor, and then obtain the proportion of variance explained by the first factor (principal component). If the proportion of variance explained by the first factor exceeds 50%, there may be a serious common method bias. In our data, for each scale, the variance explained percentage does not exceed 30%, indicating there is no serious common method bias that may largely affect the conclusion.

### 2.5 Severity of psychological problems

The students were classified according to the diagnostic criteria in Table 1, based on the results of the psychological health survey. All subsequent investigations in this study were conducted based on those criteria.

**Table 1:** Classification of the severity of psychological problems, where * represents that the respondent is considered to be in a state of illness.

**Scale**	**Total score**	**Symptom**	**Criterion**
SDQ-S	40	0-Healthy	0 ≤ score ≤ 15
		1-Moderate*	16 ≤ score ≤ 19
		2-Heavy*	20 ≤ score ≤ 40
PQ-16	48	0-No risk of psychosis	Score ≥ 10
		1-Risk for psychosis*	Score < 10
GAD-7	21	0-No anxiety	0 ≤ score < 5
		1-Mild anxiety*	5 ≤ score < 10
		2-Moderate anxiety*	10 ≤ score < 15
		3-Severe anxiety*	15 ≤ score
CRIES-13	65	0-Healthy	Score ≤ 17
		1-Moderate*	18 ≤ score ≤ 31
		2-Heavy*	32 ≤ score
PSQI	21	0-Very good sleep quality	Score = 0
		1-Fairly good sleep quality	1 ≤ score < 11
		2-Poor sleep quality*	11 ≤ score < 16
		3-Very poor sleep quality*	16 ≤ score
IGDS9-SF	45	0-No internet addiction	Questions that scores 5 points < 5
		1-Internet addiction*	Question that scores 5 points ≥ 5
PHQ-9	27	0-Healthy	0 ≤ score < 5
		1-Mild depression*	5 ≤ score < 10
		2-Moderate depression*	10 ≤ score < 15
		3-Moderately severe depression*	15 ≤ score < 20
		4-Severe depression*	20 ≤ score
WEMWBS	70	0-Very low level of mental health*	14 ≤ score < 32
		1-Low level of mental health*	32 ≤ score < 40
		2-Moderate level of mental health	40 ≤ score < 59
		3-Very high level of mental health	59 ≤ score ≤ 70

The regression analysis in Section 3.6 below is based on this dichotomy of “ill—not ill”.

### 2.6 Statistical methods

Based on the classification of psychological problems, as described above, we performed chi-square tests to validate the significance of the correlations between different variables, including the correlations between school bullying and the presence of psychological problems and between the degree of bullying and the presence of psychological problems. After confirming the correlations between school bullying and psychological problems, we further performed logistic regression to quantitatively analyze those correlations. For all the results from the statistical analyses, a *p*-value < 0.05 was considered to indicate the statistical significance.

## 3 Results

### 3.1 Overview of school bullying

**Figure 1:**
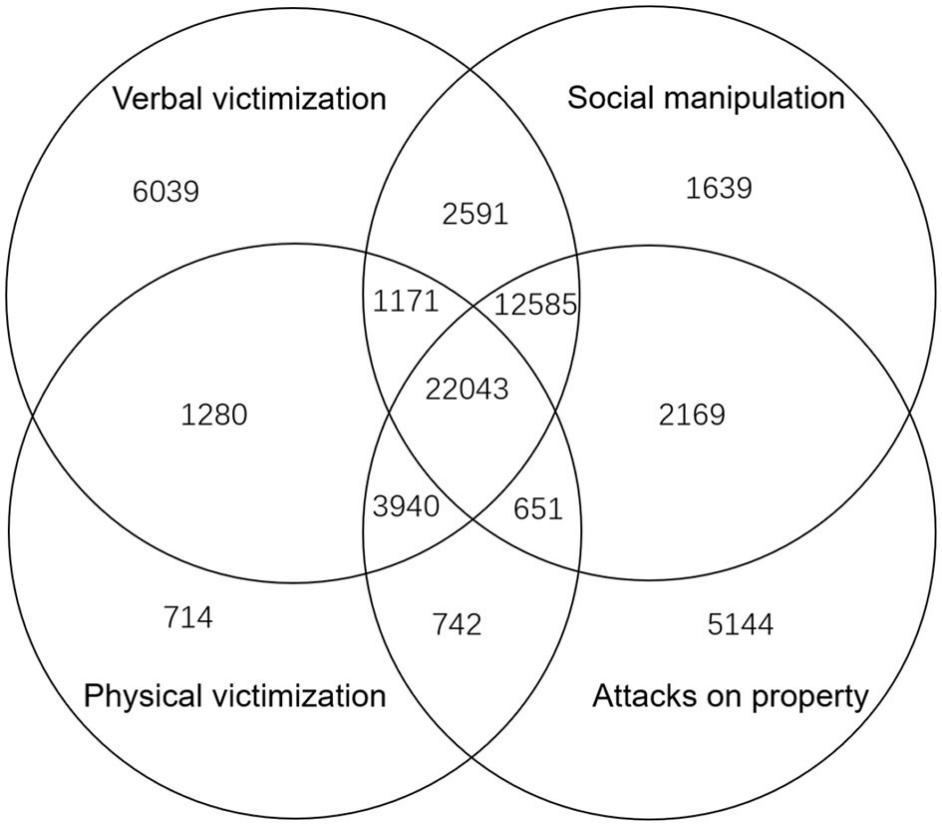
A Venn diagram of types of school bullying. The Venn diagram illustrates the number of students who have experienced different types of school bullying. Each block is marked by the number of students in the corresponding set. For example, 1,171 represents the number of students who have experienced verbal victimization, physical victimization, and social manipulation, but not attacks on property. Two data points are not shown in the diagram: 7,455 students who experienced both verbal victimization and attacks on property and 179 students who experienced both physical victimization and social manipulation.

Of the 95,545 primary and secondary school students surveyed, 68,342 (71.6%) reported that they experienced school bullying of varying types and degrees. Based on the MPVS scores, we classified students who experienced school bullying into two categories by the degree of victimization: mild victimization (MPVS score ≤ 16) and severe victimization (MPVS score larger than 16). Among those who experienced school bullying, 56,372 and 11,970 students experienced mild and severe bullying, accounting for 59 and 12.5% of the total sample, respectively (see Table 2). Table 2 reveals that there are overlaps among different types of bullying, namely some students may have experienced multiple types of bullying, as indicated in Figure 1.

**Table 2:** The overall statistics of school bullying in this survey.

	**Category**	**Samples**	**Percentage**
Whether being bullying	Being bullied	68,342	71.6%
	Not being bullied	27,113	28.4%
Types of school bullying	Physical victimization	30,720	32.2%
	Verbal victimization	57,104	59.8%
	Social manipulation	43,028	45.10%
	Attacks on property	54,729	57.30%
Severity of school bullying	Mild bullying	56,372	59.00%
	Evere bullying	11,970	12.50%

### 3.2 Overall impact of being bullied

Table 3 lists the eight dimensions of psychological problems. Among all participants, the prevalence of severe emotional and behavioral problems (SDQ-S) was 11.4%; 16.5% were found to be at risk of mental illness (PQ-16); 12.9% reported moderate to severe anxiety (GAD-7); 22.6% showed relatively strong stress responses, with 4.9% at risk of developing PTSD (CRIES-13); 6.1% of students had poor or very poor sleep quality (PSQI); 7.7% experienced internet addiction (IGDS9-SF); 16.2% had moderate to severe depression (PHQ-9); and 28.1% had low or very low levels of mental health (WEMWBS). These findings indicate that the psychological conditions of Chinese primary and secondary school students are poor as a whole. The results of the chi-square χ^2^-tests showed significant correlations between experiencing bullying and all eight psychological problems, that is, students who were bullied had significantly poorer psychological conditions than those who were not.

**Table 3:** The impact of school bullying on psychological conditions (*: <0.1, **: <0.05, ***: <0.01, the same below).

**Psychological dimensions**	**Symptom codes**	**All**	**Not being bullied**	**Being bullied**	**χ^2^**	***P*-value**
SDQ-S	0	70,848 (74.2%)	24,288 (89.6%)	46,560 (68.1%)	13.9	***
	1	13,754 (14.4%)	1,774 (6.5%)	11,980 (17.5%)		
	2	10,853 (11.4%)	1,051 (3.9%)	9,802 (14.3%)		
PQ-16	0	79,744 (83.5%)	25,892 (95.5%)	53,825 (78.8%)	17.9	***
	1	15,711 (16.5%)	1,221 (4.5%)	14,490 (21.2%)		
GAD-7	0	48,749 (51.1%)	21,491 (79.3%)	27,258 (39.9%)	33.4	***
	1	34,374 (36.0%)	4,841 (17.9%)	29,533 (43.2%		
	2	9,101 (9.5%)	612 (2.3%)	8,489 (12.4%)		
	3	3,231 (3.4%)	169 (0.6%)	3,067 (4.5%)		
CRIES-13	0	73,861 (77.4%)	24,857 (91.7%)	49,004 (71.7%)	16.3	***
	1	16,932 (17.7%)	1,868 (6.9%)	15,064 (22.0%)		
	2	4,661 (4.9%)	387 (1.4%)	4,274 (6.3%)		
PSQI	0	64,312 (67.4%)	23,798 (87.8%)	40,514 (59.3%)	21.0	***
	1	25,326 (26.5%)	2,884 (10.6%)	22,442 (32.8%)		
	2	5,366 (5.6%)	395 (1.5%)	4,971 (7.3%)		
	3	451 (0.5%)	36 (0.1%)	415 (0.6%)		
IGDS9-SF	0	88,093 (92.3%)	26,468 (97.6%)	61,625 (90.2%)	4.7	**
	1	7,362 (7.7%)	654 (2.4%)	6,717 (9.8%)		
PHQ-9	0	56,108 (58.8%)	21,649 (79.8%)	34,459 (50.4%)	19.4	***
	1	23,786 (24.9%)	4,227 (15.6%)	19,559 (28.6%)		
	2	9,963 (10.4%)	877 (3.2%)	9,086 (13.3%)		
	3	3,838 (4.0%)	269 (1.0%)	3,569 (5.2%)		
	4	1,760 (1.8%)	91 (0.3%)	1,669 (2.4%)		
WEMWBS	0	12,385 (13.0%)	2,671 (9.9%)	9,714 (14.2%)	20.2	***
	1	14,367 (15.1%)	1,715 (6.3%)	12,652 (18.5%)		
	2	48,620 (50.9%)	11,995 (44.3%)	36,625 (53.6%)		
	3	20,083 (21.0%)	10,732 (39.6%)	9,351 (13.7%)		

### 3.3 The impact of bullying severity

After establishing significant correlations between bullying and mental health, we further analyzed correlations between the bullying severity and the presence of psychological problems. As shown in Table 4, students who experienced mild bullying and students who experienced severe bullying significantly differed in terms of psychological conditions. The chi-square χ^2^-tests revealed that the severity of bullying is significantly correlated with seven psychological dimensions, except for the mental well-being, demonstrating that a higher severity of bullying experiences is associated with poorer psychological conditions.

**Table 4:** The impact of the severity of bullying on psychological conditions.

**Psychological dimensions**	**Symptom codes**	**Bullied**	**Mildly bullied**	**Severely bullied**	**χ^2^**	***P*-value**
SDQ-S	0	46,560 (68.1%)	41,401 (73.4%)	5,159 (43.1%)	20.8	***
	1	11,980 (17.5%)	9,045 (16.0%)	2,935 (24.5%)		
	2	9,802 (14.3%)	5,926 (10.5%)	3,876 (32.4%)		
PQ-16	0	53,825 (78.8%)	47,300 (83.9%)	6,552 (54.7%)	20	***
	1	14,490 (21.2%)	9,072 (16.1%)	5,418 (45.3%)		
GAD-7	0	27,258 (39.9%)	25,210 (44.7%)	2,048 (17.1%)	25.1	***
	1	29,533 (43.2%)	24,053 (42.7%)	5,479 (45.8%)		
	2	8,489 (12.4%)	5,596 (9.9%)	2,893 (24.2%)		
	3	3,067 (4.5%)	1,512 (2.7%)	1,550 (12.9%)		
CRIES-13	0	49,004 (71.7%)	43,419 (77.0%)	5,585 (46.7%)	21.1	***
	1	15,064 (22.0%)	10,767 (19.1%)	4,297 (35.9%)		
	2	4,274 (6.3%)	2,186 (3.9%)	2,088 (17.4%)		
PSQI	0	40,514 (59.3%)	35,957 (63.8%)	4,557 (38.1%)	16	**
	1	22,442 (32.8%)	17,299 (30.7%)	5,145 (43.0%)		
	2	4,971 (7.3%)	2,957 (5.2%)	2,014 (16.8%)		
	3	415(0.6%)	159(0.3%)	256(2.1%)		
IGDS9-SF	0	61,625 (90.2%)	52,370 (92.9%)	9,255 (77.3%)	9.5	**
	1	6,717 (9.8%)	4,002 (7.1%)	2,715 (22.7%)		
PHQ-9	0	34,459 (50.4%)	29,364 (52.1%)	5,095 (42.6%)	10.2	**
	1	19,559 (28.6%)	17,020 (30.2%)	2,539 (21.2%)		
	2	9,086 (13.3%)	6,856 (12.2%)	2,230 (18.6%)		
	3	3,569 (5.2%)	2,321 (4.1%)	1,248 (10.4%)		
	4	1,669 (2.4%)	811 (1.4%)	858 (7.2%)		
WEMWBS	0	9,714 (14.2%)	6,761 (12.0%)	2,953 (24.7%)	7.2	*
	1	12,652 (18.5%)	9,949 (17.6%)	2,703 (22.6%)		
	2	36,625 (53.6%)	31,478 (55.8%)	5,147 (43.0%)		
	3	9,351 (13.7%)	8,184 (14.5%)	1,167 (9.7%)		

To summarize the results from Tables 3, 4, students who experienced school bullying had generally poorer psychological conditions and a significantly higher risk of developing mental illnesses compared to students who did not experience it. For example, the proportion of students at risk of prodromal psychosis was 4.5% among those who never experienced school bullying, while it was 21.2% among those who experienced it, with 16.1 and 45.3% for those who experienced mild and severe bullying, respectively. All dimensions of psychological problems, with the exception of mental wellbeing, showed a consistent pattern of a higher risk of developing mental illness in those who experienced more severe bullying.

### 3.4 The impact of different types of bullying

Among all participants, 30,720, 57,104, 43,028, and 54,729 subjects reported experiencing physical victimization, verbal victimization, social manipulation, and attacks on property, respectively, accounting for 44.9, 83.6, 73.7, and 80% of all victims. Some participants reported experiencing multiple types of bullying. However, as shown in Table 5, there was no significant difference in the psychological conditions of victims in terms of the type of bullying. The chi-square χ^2^-tests confirmed no significant correlation between the type of school bullying and psychological conditions. In a word, the occurrence of psychological problems among victims is related to the extent of their exposure to bullying but not the type of bullying.

**Table 5:** The impact of different types of bullying on psychological problems.

**Psychological dimensions**	**Symptom codes**	**All**	**TypeA**	**TypeB**	**TypeC**	**TypeD**	**χ^2^**	***P*-value**
SDQ-S	0	70,848 (74.2%)	17,887 (58.2%)	37,145 (65.0%)	25,930 (60.3%)	35,923 (65.6%)	1.72	0.94
	1	13,754 (14.4%)	6,567 (21.4%)	10,714 (18.8%)	8,918 (20.7%)	10,072 (18.4%)		
	2	10,853 (11.4%)	6,266 (20.4%)	9,245 (16.2%)	8,180 (19.0%)	8,734 (16.0%)		
PQ-16	0	79,744 (83.5%)	21,399 (69.7%)	43,567 (76.3%)	30,094 (71.9%)	41,736 (76.3%)	1.67	0.64
	1	15,711 (16.5%)	9,321 (30.3%)	13,537 (23.7%)	12,094 (28.1%)	12,993 (23.7%)		
GAD-7	0	48,749 (51.1%)	9,515 (31.0%)	20,713 (36.3%)	13,065 (30.4%)	19,685 (36.0%)	1.54	>0.99
	1	34,374 (36.0%)	14,214 (46.3%)	25,630 (44.9%)	20,322 (47.2%)	24,691 (45.1%)		
	2	9,101 (9.5%)	5,006 (16.3%)	7,859 (13.8%)	6,972 (16.2%)	7,532 (17.2%)		
	3	3,231 (3.4%)	1,985 (6.5%)	2,902 (5.1%)	2,669 (6.2%)	2,821 (5.2%)		
CRIES-13	0	73,861 (77.4%)	19,019 (61.9%)	39,285 (68.8%)	27,645 (64.2%)	37,705 (68.9%)	1.96	>0.99
	1	16,932 (17.7%)	8,664 (28.2%)	13,735 (24.1%)	11,631 (27.0%)	13,129 (24.0%)		
	2	4,661 (4.9%)	3,037 (9.9%)	4,084 (7.2%)	3,752 (8.7%)	3,895 (7.1%)		
PSQI	0	64,312 (67.4%)	15,795 (51.4%)	32,281 (56.5%)	22,087 (51.3%)	30,625 (56.0%)	1.16	>0.99
	1	25,326 (26.5%)	11,439 (37.2%)	19,785 (34.6%)	16,347 (38.0%)	19,204 (35.1%)		
	2	5,366 (5.6%)	3,168 (10.3%)	4,637 (8.1%)	4,211 (9.8%)	4,509 (8.2%)		
	3	451 (0.5%)	318 (1.0%)	401 (0.7%)	383 (0.9%)	391 (0.7%)		
IGDS9-SF	0	88,093 (92.3%)	26,148 (85.1%)	50,765 (88.9%)	37,451 (87.0%)	48,696 (89.0%)	0.93	0.81
	1	7,362 (7.7%)	4,572 (14.9%)	6,339 (11.1%)	5,577 (13.0%)	6,023 (11.0%)		
PHQ-9	0	56,108 (58.8%)	15,363 (50.0%)	27,628 (48.4%)	19,527 (45.4%)	26,135 (47.8%)	1.04	1
	1	23,786 (24.9%)	7,709 (25.1%)	16,399 (28.7%)	12,195 (28.3%)	16,003 (29.2%)		
	2	9,963 (10.4%)	4,486 (14.6%)	8,178 (14.3%)	6,921 (16.1%)	7,867 (14.4%)		
	3	3,838 (4.0%)	2,062 (6.7%)	3,313 (5.8%)	2,914 (6.8%)	3,179 (5.8%)		
	4	1,760 (1.8%)	1,100 (3.6%)	1,586 (2.8%)	1,471 (3.4%)	1,545 (2.8%)		
WEMWBS	0	12,385 (13.0%)	5,758 (18.7%)	8,804 (15.4%)	7,449 (17.3%)	8,160 (14.9%)	1.15	>0.99
	1	14,367 (15.1%)	6,525 (21.2%)	11,187 (19.6%)	9,192 (21.4%)	10,658 (19.5%)		
	2	48,620 (50.9%)	15,084 (49.1%)	30,026 (52.6%)	21,881 (50.9%)	29,173 (53.3%)		
	3	20,083 (21.0%)	3,353 (10.9%)	7,087 (12.4%)	4,508 (10.5%)	6,738 (12.3%)		

TypeA, Physical victimization; TypeB, Verbal victimization; TypeC, Social manipulation; TypeD, Attacks on property.

### 3.5 Differences in school bullying at different grade levels

Based on the grade levels (primary, junior high, or senior high school) of the students, we analyzed the differences in the proportion of students who experienced school bullying, as well as the types and degrees of bullying. As shown in Table 6, the proportions of students in primary school, junior high school, and high school who experienced school bullying were 71.6, 67.4, and 71.8%, respectively. As confirmed by the chi-square χ^2^-tests, there were no significant differences between the three groups.

**Table 6:** The difference in bullying prevalence among different grade levels.

	**Category**	**Members**	**Primary school**	**Junior high school**	**Senior high school**	**χ^2^**	***P*-value**
Whether being bulling	Being bullied	68,342 (71.6%)	18,271 (67.4%)	31,964 (74.1%)	18,107 (71.8%)	1.1	0.56
	Not being bullied	27,113 (28.4%)	8,857 (32.6%)	11,160 (25.9%)	7,096 (28.2%)		
Types of bullying	Physical victimization	30,720 (32.2%)	16,470 (60.7%)	13,923 (32.3%)	6,139 (24.4%)	16	**
	Verbal victimization	57,104 (59.8%)	15,530 (57.2%)	27,194 (63.1%)	14,380 (57.1%)		
	Social manipulation	43,028 (45.1%)	12,327 (45.4%)	20,080 (46.6%)	10,621 (42.1%)		
	Attacks on property	54,729 (57.3%)	14,495 (53.4%)	25,663 (59.5%)	14,571 (57.8%)		
Severity of bullying	Mild bullying	56,372 (82.5%)	14,160 (77.5%)	26,526 (83%)	15,956 (88.1%)	3.9	**
	Severe bullying	11,970 (17.5%)	4,111 (22.5%)	5,708 (17%)	2,151 (11.9%)		

In primary school, 16,470 (60.7%) students experienced physical victimization. In contrast, the proportions of such students in junior high school and high school were significantly lower, at 32.3 and 24.4%, respectively. A significantly higher proportion of primary school students experienced severe bullying than junior high school and senior high school students, and a significantly higher proportion of junior high school students experienced severe bullying than senior high school students. The chi-square χ^2^-tests validated the statistical significance of the above two correlations. Based on these findings, we recommend paying particular attention to school bullying incidents in primary schools, especially those involving physical victimization.

### 3.6 Relationship between school bullying and the probability of psychological illnesses

Having established the significant correlations between school bullying experience and psychological problems, we sought to demonstrate the impact of school bullying experience on the risk of developing psychological illnesses in a more intuitive way. To achieve this, we divided the participants into two groups: those with and those without the psychological illness for each dimension, according to Table 1. Next, we performed a logistic regression on each dimension. Table 7 presents the results, with the odds ratio indicating the likelihood of having the corresponding psychological illness for the group that experienced school bullying compared to the group that did not. For example, as shown in Table 7, the odds of developing emotional and behavioral disorders for students who experienced mild and severe school bullying were 3.10 and 11.35 times higher, respectively, than for students who did not experience bullying. These results indicate that experiencing school bullying significantly increases the probability of developing psychological illnesses, and this probability increases with the severity of bullying.

**Table 7:** Regression results of the 8 dimensions of psychological problems.

**Psychological dimensions**	**Severity**	**Reference value**	**Odds ratio**	**95% confidence interval**	***P*-value**
SDQ-S	Mild bullying	No bullying	3.10	2.97–3.24	***
	Severe bullying		11.35	10.76–11.97	***
PQ-16	Mild bullying	No bullying	4.06	3.82–4.32	***
	Severe bullying		17.53	16.38–18.76	***
GAD-7	Mild bullying	No bullying	4.72	4.56–4.88	***
	Severe bullying		18.52	17.51–19.58	***
CRIES-13	Mild bullying	No bullying	3.28	3.13–3.44	***
	Severe bullying		12.59	11.90–13.32	***
PSQI	Mild bullying	No bullying	4.07	3.91–4.24	***
	Severe bullying		11.67	11.08–12.29	***
IGDS9-SF	Mild bullying	No bullying	3.13	2.88–3.41	***
	Severe bullying		12.03	11.01–13.15	***
PHQ-9	Mild bullying	No bullying	3.64	3.52–3.77	***
	Severe bullying		5.34	5.10–5.60	***
WEMWBS	Mild bullying	No bullying	2.18	2.10–2.26	***
	Severe bullying		4.64	4.42–4.87	***

## 4 Conclusion and discussion

In this study, we investigated the status of school bullying among primary and secondary school students in Zigong City. The results showed that of the 95,545 surveyed students, 71.6% (*68,342*) experienced school bullying of varying degrees, and among students who were bullied, 17.5% (*11,970*) experienced severe bullying. This indicates that school bullying is a prevalent issue among primary and secondary school students, with a higher incidence among the former. Additionally, the severity is more pronounced among primary school students than their secondary school counterparts. There are notable differences in the prevalence of school bullying across different countries. In a previous study, the percentage of students involved in school bullying across different countries ranged from 6.3 to 45.2% (Craig et al., 2009). In comparison, our investigation reported much higher percentage. This difference may be attributed to the relatively higher levels of social development and implementation of preventive laws and regulations against school bullying in the European countries. Meanwhile, China, as a developing country, and Zigong, as an underdeveloped city in China, need more efforts to address this issue.

The chi-square tests and logistic regression showed that the occurrence and severity of school bullying are significantly correlated with all eight psychological dimensions. Compared with students who never experienced school bullying, students who experienced school bullying were more likely to have psychological problems, and the severity of bullying was positively correlated with a higher likelihood of psychological problems. These findings are consistent with previous research, which suggests that victims of school bullying may struggle to solve life problems and often have negative attitudes and poor interpersonal relationships (Esposito et al., 2019). Other studies indicate that the social pressure experienced by victims may result in a strong sense of threat, which may lead to psychological problems such as depression, anxiety, fear of attending school, and feelings of insecurity and dissatisfaction in school (Moyano and del Mar Sanchez-Fuentes, 2020). Additionally, the vigilance and stress response of teenagers during puberty may increase significantly, potentially increasing the risk of psychological problems in the conflicting environment (Plexousakis et al., 2019).

Studying the relationship between school bullying and psychological conditions has important social value and practical significance. It provides a better understanding of the origin of school bullying and how school bullying impacts students' mental health, thereby offering a scientific basis for preventing and reducing school bullying. It also helps in treating bullying-related psychological problems.

School bullying is a serious social problem that considerably affects the physical and mental health of victims, leading to emotional and behavioral problems, anxiety, depression, PTSD, and even extreme behaviors such as suicide. To reduce the occurrence of school bullying, we propose the following suggestions. First, school administrators, teachers, and parents should dearly understand what bullying is and its impacts on the physical and mental health. They should strengthen education on preventing bullying, closely monitor students, and pay attention to students' daily lives. Second, both society and schools should take bullying seriously, promptly stop bullying incidents, and provide necessary help to students with psychological problems. Thirdly, after a bullying incident occurs, school administrators and parents should immediately pay attention to the safety of the victims, dig out the reason leading to the bullying incident, figure out the detailed process and property of the bullying incident, help the victims overcome their psychological trauma, improve their self-protection abilities, and prevent further occurrences. Fourth, educational managers and school administrators should prioritize creating a safer and more inclusive campus. Preventing school bullying should be a core part of developing a safe school. Technological means, routine surveys, and trained inquiry methods should be used to promptly identify and address potential school bullying incidents.

The present study has several strengths worth mentioning. First, the sample size was large, with 95,545 students surveyed, providing significant and stable statistical results. Second, the study was conducted in a developing country, making the findings more relevant to other developing countries. Third, the study differentiated between the four types and the severity of school bullying, and thus drew detailed conclusions. Finally, the study utilized multiple scales and questionnaires to measure various psychological problems, allowing for a comprehensive analysis of the quantitative relationships between school bullying and various psychological illnesses.

This study also has certain limitations. Firstly, the cross-sectional design precludes causal inferences about the relationship between school bullying and psychological problems and only suggests correlations. The focus of our future research should be on establishing causal relationships between school bullying and psychological problems through longitudinal studies. Secondly, the data were self-reported by the students, which may have subjective biases, potentially overestimating or underestimating the correlations. Future research should employ more objective measures to collect data on students' psychological issues and bullying behaviors. Thirdly, as shown in Figure 1, many students suffer multiple types of bullying. However, in this paper we only consider the roles of whether being bullied, the severity of bullying and the different types of bullying. In the future work, we will analyze what will happen if a student suffer multiple types of bullying. To do so, we need more samples to ensure the statistical significance. Fourthly, our research focused solely on a specific city, and while it may have some representativeness, the comparative analysis of other Chinese cities during the same period is insufficient. Finally, it is worth noting that the occurrence of school bullying and psychological illnesses may not be evenly distributed among the population, which may have led to skewed probability estimates. Moreover, the COVID-19 pandemic may have largely impacted the findings. Therefore, future studies should consider more in-depth comparative analysis, using data from post-pandemic investigations, to provide a more accurate understanding of the relationship between school bullying and psychological problems among school students.

## Data availability statement

The raw data supporting this study can be obtained on request to the corresponding author, provided that the data will not be used for commercial purposes.

## Ethics statement

The studies involving humans were approved by the Ethics Committee of University of Electronic Science and Technology of China. The studies were conducted in accordance with the local legislation and institutional requirements. Written informed consent for participation in this study was provided by the participants' legal guardians/next of kin.

## Author contributions

NZ: Conceptualization, Investigation, Project administration, Writing—original draft. SY: Investigation, Writing—original draft. QZ: Investigation, Writing—review & editing. JW: Writing—original draft. WX: Data curation, Resources, Writing—review & editing. YT: Data curation, Resources, Writing—review & editing. TZ: Conceptualization, Funding acquisition, Investigation, Methodology, Supervision, Writing— original draft.
